# A long journey to the discovery of nuclear receptor coactivator existence, physiology, pathology, and therapy

**DOI:** 10.1016/j.jbc.2025.108415

**Published:** 2025-03-14

**Authors:** Bert W. O'Malley

My early life began with a wonderful set of parents in a poor section of Pittsburgh. I was educated in local Catholic schools until I entered the University of Pittsburgh on scholarship as a major in psychology and chemistry. I had a growing interest in a career in medicine promoted to me by my mother. It was at Pitt that I was lucky enough to meet and marry Pitt’s Homecoming Queen and my beautiful partner for life, Sally. We quickly had two children. Our finances did not allow me to go to an Ivy League medical school, so I entered Pitt and worked part-time afternoons in a biochemistry lab that investigated adrenal steroid hormone synthesis; I also worked at a local hospital in the evenings, simply for family income. At that time, I was not interested in a career in research. I did very well in medical school and was advised by my brilliant chief of medicine (Jack Myers) to do my internship/residency at Duke University under the tutelage of the nationally admired Eugene Stead. Jack and Gene had been together at the Brigham in a group of famous physicians who went on to populate medical schools and modern academic hospitals across the country. At the time, Stead was building a new academic powerhouse at Duke Hospital with a new protocol for referred patients and a very rigorous training program for interns/residents. I became convinced that despite being in a small rural North Carolina town, it was one of the best places in the country for advanced medical training. Despite the shock to my wife and me when we found that my starting salary would be $37.50/month, it was the best choice I could have made for my clinical training. The Duke work schedule was brutal (17 h per day for six and a half days per week). Even then, I had to again work part time afternoons in a hematologic biochemistry lab to barely make ends meet.

Although I now had over 7 years of experience in lab research, I still imagined my career to be as a physician, not a scientist. We worked “like dogs” at Duke for 2 years, but Sally supported me emotionally while taking care of the kids and the house. Like many medical students in the era of the Vietnam War, after my second year at Duke, I chose to join the Navy and, as a doctor, was relocated to the National Institute of Health (NIH) as a Clinical Associate. During those years, NIH was the absolute pinnacle of U.S. science and served as the training ground that provided for the subsequent explosive development of research faculty in U.S. medical schools. I obtained a fellowship position in the Endocrine Branch of the National Cancer Institute. We did clinical work in the morning and research from noon to midevening. It was fun work and I now had a lifesaving government salary of $13,000/year. With a V.A. loan, I was even able to put a down payment on a house for my wife and now four kids.

I needed a lab project and chose to work with a bright young faculty member (Stan Korenman) on the chicken oviduct, which had been identified to produce estrogen-induced mucous secretions (egg white protein) by Roy Hertz some years before in endocrine branch. I was an eager trainee with Stan. He made antibodies to the egg white proteins (ovalbumin and avidin) and I developed an *in vitro* tissue synthesis system where progesterone (PR)/estrogen induced avidin and ovalbumin proteins. However, Stan suddenly was offered a good job in Los Angeles and left NIH.

Since I was the only scientist left in that lab and the work was gaining notoriety, Mort Lipsett offered me a position as lab head in my second year. Although young, I had an idea that steroid hormones worked by inducing the egg white proteins via mRNA intermediates. I published my hypothesis that steroid hormones induced new DNA-dependent mRNAs. I guess I was enamored with the Jacob-Monod theory in bacteria. Although an obvious explanation nowadays, it was not then. My enthusiasm was tempered by the lack of support for my mRNA theory by senior luminaries in the field of hormone action. One published review in a top journal even questioned the existence of mRNAs. A large variety of theories on hormone stimulatory mechanisms were promulgated at that time, including 1) membrane induced transport of precursor nucleotides and amino acids; 2) induction of second messenger cAMP; 3) ribosomal stimulation of peptide synthesis; 4) mRNA stabilization from degradation; 5) posttranscriptional processing of mRNA; and 6) our theory of transcriptional mRNA induction (only proffered by one other, the Kenny lab at Oak Ridge National Labs). Nevertheless, I persevered in my hypothesis despite the pushback.

Since the technology to isolate mRNA was unknown, I first used indirect methodology to show an effect of hormones on the nuclear transcription system. Bill McGuire and I turned to nearest-neighbor analysis and DNA-RNA hybridization technology to show that new “species” of nuclear RNA were synthesized in response to steroid hormones. I believed our results supported my theory that steroid hormones were acting at the transcriptional level on DNA and that these newly hormone-induced transcripts likely contained specific mRNAs.

At this point in time, I received a number of job offers to move to universities. I turned all of them down but found that an offer by Grant Liddle at Vanderbilt University was becoming hard to ignore. After 6 months of saying no, their offer got up to an appointment as an Endowed Birch Professor to form a new Center for Reproductive Hormone Action. At this point, my Branch head Lipsett told me this was too good to turn down and that I should accept the offer. Just before leaving the NIH, I had hired Tony Means into my unit and he moved with me. Off to Music City, the family went for new adventures.

Science at Vanderbilt at that time was superb and it was there that Tony and I delved deeply into the mechanisms of hormone action. I was committed to determine once and for all whether steroid receptors indeed turned on new synthesis of specific mRNAs to increase the levels of specific proteins. Yet, proving this idea required advanced methodologies in protein chemistry and RNA biology. Tony Means, Gary Rosenfeld, Jeff Rosen, and I (together with a couple postdocs) soon demonstrated that estrogen indeed induced new oviduct ovalbumin mRNA synthesis using a translatable ribosome system; we concluded that newly induced mRNA coded for new ovalbumin protein synthesis. Thus, we completed the first demonstration of the primary pathway of hormone action: from steroid to receptor to DNA to ovalbumin mRNA to ovalbumin protein to function (1). This was the lab’s sentinel early achievement. However, the mechanism soon got more complex. Enter coactivators!

Our lab’s discovery of the elusive and mysterious coactivators began in 1970 when my colleague Bill Schrader purified one of the first nuclear receptors (NRs) (for PR) (2). Our lab had developed a new and reliable cell-free transcription system to assess receptor transcriptional activity. At that time, our available cell/tissue data obtained with Tony Means led us to the conclusion that PR and estrogen receptors (ERs) acted as DNA-dependent transcriptional regulators to induce synthesis of new mRNAs and proteins in oviduct cells (1). Since there were no ovalbumin and avidin mRNAs before hormone administration, we believed the mRNAs had to be newly induced, implicating action at the DNA level, likely via their cellular NRs.

When we used Bill Schrader’s purified PR, we found that a conformational change in receptor structure was induced upon hormone binding, while a distinctly different conformation was induced by hormone antagonists (3). We felt this was how receptors were armed for function. However, highly purified PR complexed with hormone bound only weakly to DNA, and it had no intrinsic transcription stimulatory activity when added into our chromatin transcription system. Although purification by various means yielded no active receptor, we noted that crude preparations of PR had increased DNA binding and slight transcriptional stimulatory activity. We reasoned that this result likely occurred because there was a missing associated “nuclear” factor that was needed for receptor-mediated transcription. Thus began our search for the missing factor using crude nuclear extracts reconstituted with DNA and purified PR. Tom Spelsberg became the key player in the next phase of our chromatin experiments. Our reconstitution experiments with pure PR, DNA, and polymerase along with Tom’s partially purified nuclear protein fractions soon led us to conclude that the missing ingredient was not histones, but a type of unknown nuclear non-histone “adapter protein” (4). It bound not to the DNA, but to the receptor, and increased its affinity for DNA and its ability to enhance DNA-dependent RNA synthesis. We initially, and mistakenly, believed that there was likely to be “only one” of these missing adaptor proteins that we then termed “acceptor protein” (5).

Since, at that time, we were using only partially purified, non-histone preparations in our experiments, this experimental setup left openings for doubt and alternative explanations by other scientists. We accepted the challenge to purify the missing adaptor protein to prove our hypothesis more conclusively. Despite our prowess at protein purifications, we failed in this task; our protein fractionations when subjected to multiple columns and protocols produced receptor-stimulating activities ranging over a broad range of protein sizes and charges. At that time, the explanation for this broad range of diverse activities was unknown and confusing to us. So, after a year of periodic failed attempts, we backed off this purification task—but I did not disbelieve our hypothesis. It was not until over a decade later in the early 1990s that we returned to this project.

Means, Schrader and I dedicated most of the 1970s in obtaining definitive proof that NRs actually were indeed transcription factors (TFs) that could induce the synthesis of specific mRNAs and protein. The field expanded significantly with Greene and Jensen’s purification of ER, Brad Thompson’s purification of glucocorticoid receptor and Bill Schrader’s purification of PR (2); Bill showed that there were two forms of PR (termed A and B), each having different physiologies. Again, significant controversy ensued in our field over the A/B forms, but our results were proven to be correct. The lab carried out additional experiments to show how a hormone activated a receptor’s conformation and showed it could be activated in the “absence of ligand” by growth factor pathway-induced phosphorylation.

We employed our chick oviduct transcription system with Ming and Sophia Tsai, and demonstrated that estrogen/PR initiated the *de novo* synthesis of ovalbumin and avidin mRNAs and proteins in cell-free, cell culture and *in vivo* animal model systems (6). Our experiments on receptors and hormone action were new and not easily accepted by the luminaries in the nascent field of hormone action. I remained fixated on induction of DNA-mediated new mRNA synthesis as the most logical explanation of hormone action (1). It was clear to me that hormone-activated receptors changed not just the amount of nuclear RNA, but the specific types of mRNAs, which contained new mRNAs.

It was at this point that we moved the lab again, this time to the Baylor College of Medicine (BCM) in Houston. We had outgrown our space over time in Nashville and we all agreed a move was in our future. I looked at offers from Harvard and Rockefeller that were attractive, but BCM allowed me to set up a brand new department of Cell Biology, one of the first in the country. It also allowed me to bring a large number of our group and provided adequate seed funding, space, and positions. After a few visits to BCM and discussions with its President Michael DeBakey, I decided to relocate to Houston. We brought seven people to BCM space and a new South-Western environment. This was the time (1972) of the Vietnam War, and the whole country was undergoing a transformation and our nontraditional attitude and behavior fit right in with it. Our saving grace for the conservative BCM faculty was our high productivity, impressive generation of grant funding, and a strong backing by President DeBakey and Executive Vice President, Joe Merrill.

By the early 1980s, recombinant technology had been discovered, and our lab became adept at the earliest technology of DNA cloning (6). Ron Evans cloned glucocorticoid receptor, Pierre Chambon cloned ER, our Orla Conneely cloned PR (7) and Donald McDonnell cloned Vitamin D receptor (2, 8) in our lab. Evans’ work revealed the existence of a large and diverse superfamily of NRs.

My interest in coregulators was stimulated further by studies with Donald McDonnell (9, 10, 11, 12, 13), who discovered that positive and negative gene regulation could occur in “partnership” with yeast and mammalian protein “regulatory” molecules. We both thought long about this data and decided that they indeed implicated the existence and importance of some type of undiscovered coactivators (and perhaps corepressors). The data further substantiated my earlier conclusion that hormones act via receptors and adapter-helper molecules. I continued my RNA studies in frequent collaborations with Ming and Sophia Tsai. In the early 1990s, we finally had a breakthrough in adapter/coactivator research. My postdoc, Sergio Onate and I, in collaboration with Ming and Sophia Tsai, published the first successful cloning of one of these mysterious “acceptor proteins” (14), which we designated as steroid receptor coactivator-1 (SRC-1). The addition of our purified and newly cloned SRC-1 led to a marked enhancement of PR/ER activity in our cell-free system. Moreover, transfer of the SRC-1–containing plasmid into cells significantly increased NR-dependent stimulation of specific mRNA synthesis. This publication allowed us to define a new set of rules for future coactivator identification. However, we still needed proof for the *in vivo* physiological relevance of our coactivator. This task was accomplished by mouse genetic experiments with Jianming Xu, where we deleted the *SRC-1* gene and revealed markedly diminished uterine growth in response to estrogen administration (15). We indeed noted at the time that the molecular weight of our SRC coactivators was identical to that of Myles Brown, who found an impure fraction of nuclear protein at 160 kDa that he showed to bind to ER in a hormone-dependent manner; I believe it likely contained our SRC proteins.

We initially considered that SRC-1 might be “the only” coactivator for steroid hormone receptors but were surprised when we quickly cloned another coactivator (E6AP) (16) and then multiple others. Many other labs cloned additional coactivators, including two family relatives of the SRC-1/p160 family; (SRC-2 also called GRIP-1/TIF2) and SRC-3 (also called AIB1/ACTR/pCIP/RAC3). Richard Goodman had cloned the coactivator CBP that we later found to work in synergy with SRC proteins. Over the next 2 years, there were a dozen more NR coactivator molecules cloned in various labs. We then realized why we originally were unable to purify “the” coactivator for steroid receptors; in fact, there were many diverse coactivators of various sizes and charges, and all of them formed large functional complexes. This result explained why our earlier “NR-adaptor molecules” existed over broad size and charge ranges in our column attempts to purify “it.” Cloning of SRC-1 also led us to a new conclusion that coactivators actually functioned in large “dynamic complexes” with NRs (17) and other TFs on DNA to effect regulation of gene expression. We soon concluded that these coactivators activate virtually “all” cellular DNA-binding TFs—another new concept. We also published that coactivators are regulators of all “individual steps” of transcription by sequential recruitment of SRCs first and then other coactivators. These studies filled in some new mechanistic details in the sequence of transcription: initiation (SRCs), elongation (CARM1) (18), splicing (CoAA) (19), and ubiquitin-assisted transcription termination (E6AP) (16).

At this point, I was convinced that these mysterious molecules had much more to reveal to us. Together with Ming and Sophia Tsai, we oriented the lab to delve deeper and deeper into their mysteries. Realizing that coactivators activated virtually all of the cell’s DNA-binding TFs, I considered that mammalian coactivators evolved to coordinately activate multiple gene sets to produce their many functional physiologic responses, and that they might indeed be the long sought “master genetic regulatory molecules” (20). Of course, it is only logical that activation of all of the genes needed for a physiologic goal must occur coordinately or the response is inefficient. But, what was the best way to accomplish this temporal need; by attempting to individually activate hundreds of signals to diverse individual TFs at genes simultaneously, or rather, by activating a limited set of “master regulatory factors” that find and coordinately activate the myriad TFs required for a complex physiological goal? I decided that the latter explanation was likely the more logical explanation for the existence of coactivators. Indeed, we found that posttranslational modifications, mostly by phosphorylation codes, was the means of activating a coactivator that in turn leads to the simultaneous binding and activation of the many appropriate downstream TFs at producer genes required for expressions of a physiologic outcome response.

To complete the necessary background functional chemistry of coactivators, I felt we needed to visualize the composite structure of the coactivator-receptor-helper coactivators on DNA. To be sure of the generality of their initiation complexes, we went on to determine, together with Zhao Wang, the first distinct structures (by cryo-EM) of native receptor-coactivator transcription initiation complexes of SRCs and p300 on DNA for ER, androgen receptor, and PR (21, 22, 23).

Since we now realized the important roles of coactivators in physiology, we next considered that coactivators would likely be employed for devious purposes by diseases (24, 25). In fact, coactivators such as SRC-3 turned out to be a “one-stop shop” for malignancies to allow cancer cells to take over genetic control of the cell nuclear functions needed to achieve unrequited growth—EMT, migration, and the cancer cell’s ultimate goal of metastases. Overexpression of coactivators such as SRC-3 proved to be a hallmark of most tumors, from breast to pancreatic cancers (24). It is a major contributing mechanism for the proliferation and metastases of lethal cancer cells as well as the resistance of human cancers to multiple types of therapies (25). We even found that misexpression of coactivators could provide a basis for reproductive diseases such as endometriosis (26) and endocrine hyperplastic and metabolic diseases, and that deficiencies of coactivators produce genetic glycogen syndromes akin to that of humans (20). It seemed that these amazing molecules were not only in the midst of almost all physiologies but also in the midst of many pathologies.

At this point, perhaps I will describe my more recent academic work and life. I finally stepped down from being the department chair and accepted and assumed the title of chancellor of BCM. This is actually a great job! My main responsibility is to advise the president on affairs of BCM. I usually meet with the departmental chairs once per month and am available for meetings of faculty with problems or for those who need advice. I have limited administrative and committee paperwork. Consequently, the advantage is that I can consume myself in my own research without the multiple nagging distractions that are part of a chair’s responsibility. This was great because my research attention was needed to direct and explore a series of preclinical breakthroughs that occurred concurrently and may have significant clinical therapeutic potential for humans. First, I will describe our work on coactivator-dependent small molecule therapy.

With the large amount of data collected by our lab and others, I felt that coactivators might be ripe for regulation by small chemicals. I tried to tempt three pharmaceutical companies to attack this problem, but the answers from CEOs were largely the same; coactivator molecules were not normally designed to bind ligands for function, they were large and unstructured, and not subject to easy structural predictions. The pharma companies also did not have the in-house expertise to work with coactivators and were unwilling to hire the necessary personnel to do so, especially when they felt that such regulatory molecules were unlikely to be discovered.

Having nothing to lose after my request to Pharma’s was turned down, my colleague David Lonard offered and I agreed that our own lab group should embark on a mission to screen for and obtain functional small molecules that then might be chemically modified by Jin Wang. David’s mission was successful, and we identified small molecule drugs that inhibited SRC-coactivator function (SI1/SI2). We published a series of papers showing that they were effective inhibitors of a broad spectrum of aggressive cancers. Although these molecules were as good as most existing cancer therapy drugs, I desired something better.

In the course of this search, David found yet another surprise. He discovered a small molecule “stimulator” (MCB-613) of coactivators. I initially decided to let it lay undeveloped due to the known effects of small molecular inhibitors of coactivators on cancer cells; I feared the stimulator might activate dormant cancers in animals/humans. This concern, however, clearly turned out to be unfounded (27). I became increasingly curious as to the physiological effects of these stimulators and we embarked on diverse exploratory studies. Additional surprises were in store for us.

These coactivator stimulators were paradoxically inhibitory to cancer cell growth *in vitro* and *in vivo*. It appeared that since coactivator overexpression already was present in the cancer cell and numerous other growth genes were maximally stimulated, an additional major boost in cell stimulation by these molecules was the “straw that broke the camel’s back” in terms of cell stress. The cells underwent free-radical induced decompensation and self-directed death. Yongcheng Xu went on to chemically modify the small molecules for us to generate more potent stimulators (*e.g*., 10-1) of cell growth and repair. This work had an explosive future.

We previously observed that SRC coactivators have a required role in early mammalian fetal growth/development and found them to be useful in tissue regeneration and/or wound repair models *in vitro*. However, I felt there were “bigger fish to fry” with these SRC stimulators. Consequently, since cardiac failure was the number one cause of death in the world, we decided to first investigate their effects on damage in post myocardial infarction–induced heart failure. Lisa McClendon, in collaboration with Cliff Dacso and an experienced mouse preclinical heart surgeon (J. Martin) (28), evaluated mouse cardiac function (by ejection fraction) after tying off the left anterior descending artery. The heart failure induced cardiac ejection fraction was rapidly improved by administration with our prototype (MCB-613) coactivator stimulator drug (28). Vessel growth occurred, and fibroblast function was significantly decreased, thereby strongly inhibiting cardiac fibrosis. Cardiologists had a hard time understanding that one drug could influence multiple of these processes simultaneously. However, we knew that this effect was easily explained by the pleiotropic pathways of stimulation emanating from the massive gene regulations induced by the SRC-family in its normal coactivation function of promoting growth and repair. We then found MCB-613 also inhibited cardiac reperfusion heart damage.

We next discovered that this small molecule drug also had a positive effect on stroke recovery, markedly decreasing pathological damage and physical impairment (29). Additionally, in disease models, we noted a significant improvement in brain phosphor-Tau levels which many consider to be a causative factor in dementia and other neurodegenerative diseases. I guess the saying “*When it rains it pours*” applies well to this effects of this drug. Even reversal of central nervous system post-traumatic brain damage in the hippocampus was observed by Marc Simard in collaboration. Type-1 diabetes in an early outsourced study showed early encouraging results. Based on our exciting preclinical mouse studies, we will further develop our observations on the SRC-stimulator drug; I suspect some form of this drug will likely be in patients in the future for multiple medical applications.

A major preclinical breakthrough advance was on the horizon relative to our cell coactivator studies. In the course of earlier genetic SRC-3 whole-body KO experiments in mice, designed to evaluate its physiologic and pathologic effects, we published a collaborative experiment (2006) with J. Auwerx where we noted broad lymphocyte hyperproliferation. It was a curious observation and one that I chose to not follow up at the time. But again, I tucked the result away in the back of my mind. It was not to come to the forefront of my mind again until 2019 when we tested an animal dose response with our SRC-3 “inhibitor” drug (SI-2). Most of our prior work with this cancer inhibitor drug had been done in immune depleted severe combined immunodeficiency mice animals. Yet, when Sang Jun Han turned to the use of syngeneic animals, we obtained the equivalent inhibitory response by using only 1/10th the dose required for SCID mice. The only difference between these two genetic animal species is that their immune system is absent in the SCID mice and present in the syngeneic animals. Consequently, this result suggested to me that our cancer inhibitor drug was having some fortuitous additional therapeutic effects via the immune system. Since the main target of our drug was SRC-3, which we knew had an effect on lymphocytes from our whole-body KO experiments, it appeared that SRC-3 could be the likely perpetrator. Indeed, when we then surveyed all of the blood/immune cells for SRC-3 content, Bryan Nikolai found that regulatory T cells (Tregs) were one of the two blood cell types that contained a high level of SRC-3 (30). Since Tregs are known to normally repress the immune system’s tumor-attacking conventional T cells (Tcons), we hypothesized that our drug (SI-2) might inhibit SRC-3 in Tregs and derepress Tcons so they can attack tumors. In this way, this drug would have a dual effect, directly inhibiting tumor cell proliferation and also allowing an immune attack on the tumors. Bryan Nikolai carried out cellular studies to confirm that SI-2 could inhibit SRCs and inhibit Tcon proliferation/function significantly *in vitro* (30). However, it now was imperative to prove that SRC-3 was the major regulatory coactivator for this process for Tregs *in vivo*. To do so, Sang Jun Han created a conditional gene KO mouse with “only the src-3 gene deleted” and in “only one cell type, the Tregs.” The results again were surprising and exciting.

This study revealed that specific Treg-SRC-3–deleted mice (SRC3-KO-mice) showed lifetime reliance to injections of a variety of cancers—including aggressive triple negative breast cancer breast, prostate, melanoma, colorectal, metastatic lung, glioblastoma, and pancreatic cancers—for many of which little effective treatment of any kind exists at this time. Importantly, there was no concomitant systemic inflammation/toxicity (or cytokine storm) resulting from the Tregs-specific SRC-3 deletion (31, 32). Most cancer growths were totally eliminated for the lifetime of the animals. When early disappearing tumor sites were examined, we found a large influx of SRC-3–deleted Tregs, CD8, CD4, and natural killer antitumor cells and high concentrations of interferon-gamma (Inf-γ) and granzymes and perforin—all of which are toxic to tumors. No Inf-γ was found in the circulating blood.

Since this “apparent cure” was genetic, we devised a therapeutic approach that might be applicable to normal WT mice (and perhaps humans). We took purified SRC-3-KO-Treg cells from animals without cancer, and administered the cells to littermate animals that had an aggressive cancer. To our satisfaction, the tumors melted away in the “recipient” animals and no toxicity was observed (32). Examination of the shrinking tumors after the initial therapy revealed complete? tumor destruction and the presence of many SRC-3-KO-Tregs, CD8 cells, CD4 cells, natural killer cells, cytokines, and very high Inf-γ. However, in SRC-3-KO animals, we never found elevated circulating Inf-γ levels in serum (or spleen). Dose-response studies indicated that ∼750,000 SRC-3–deleted Tregs cells with good viability usually was needed for a therapeutic dose in mice.

Even more encouraging was the observation that these KO cell recipient mice were permanently resistant to any future cancer injections. In other words, injected SRC-3-KO-Tregs created a “vaccine-like state” in animals against future cancer. Even injections of aggressive pancreatic cancers in animals vaccinated over 275 days previously with SRC-3-KO cells were destroyed. We were able to cure pancreatic cancers under the skin, orthotopic, and even spontaneous (genetic) pancreatic cancers.

Although we expected some therapeutic advantage by deleting SRC-3 coactivator, we were astonished by the magnitude and permanence (lifetime?) of the frequent therapeutic ‘eradication’, the broad tumor-type efficacy, and the lack of any cytokine storm or even mild toxicity. The health of the animals was further verified by the fact that they lived to a normal age and could reproduce similarly to WT mice, perhaps the main criteria of a healthy animal.

For therapeutic translation of this observation to humans, we now need to *in vitro* delete SRC-3 in the Tregs from a human cancer patient, reinfuse those deleted Tregs back into the same patient, and hope to see tumor/metastasis eradication. Only then will we complete the adoptive cell therapy cycle toward a clinical “cure” for cancer. Many variations of this protocol and studies with SRC stimulators are now underway in our lab and in a company that we founded with Steve Gorlin (CoRegen founding CEO) at Baylor (33). Progress toward our therapeutic goals and Food and Drug Administration approval are underway with Cliff Dacso in our lab group leading the translational effort.

Our discovery and the elucidation of steroid hormone receptor and coactivator functions and mechanisms was a ∼60 years long and a truly exciting scientific journey for me. I have been fortunate that my excellent lab members and contributions by others in our field allowed us to accomplish such a complete body of work. We have discovered the following: a) the mechanism of hormone action at the DNA level to produce specific mRNAs and proteins; b) the biochemical conformational mechanism for “ligand activation” of NRs; c) the missing key link (coactivators) for NR transcriptional “functions”; d) the first structural mechanisms for formation of “functional NR-coactivator transcription complexes” with NRs on DNA; e) a myriad of physiological roles of coactivator functions; f) the critical “driver roles” of coactivators in many human diseases; g) small molecule chemical approaches for regulating coactivators that represent “new first-in-class approaches” to target coactivators in multiple pathologies; h) and finally, a novel adoptive cell therapy preclinical “immuno-eradication” approach that is applicable for a wide variety of cancers (33, 34). Although such broad and efficient curative results for multiple pathologies in mice have never been found previously, now it must be confirmed in humans to achieve our final goal with CoRegen-Baylor.

In reality, though, new scientific discoveries are built on the backs of predecessors. The current and preceding colleagues in my field have helped to construct the highways for future discoveries. While a young trainee at NIH in 1968, I will never forget the published hypothesis by Britten and Davidson of the likely need for some unknown “master regulators” of the genome (34). Although they mistakenly thought the regulators might be RNAs, their master regulator theory was correct in principle. Their proposal gave me an “Aha moment” that caused me to imagine how over 20,000 mammalian genes are coordinately regulated to provide physiologic functions. I realized that genes are simply analogous to the 88 keys of a piano where each key played alone produces a sound, but when played together “in combinations” produces beautiful music. Similarly, the coactivators coordinately activate the genes in combinations to produce intricate physiological goals in cells and organs. I believe that we have finally proven that coactivators are truly the “Masters of the Genome” (33, 35, 36). Unfortunately, due to their powerful natures, pathologies such as cancers frequently coopt them for their growth and functional disease purposes.

For this body of work, I am in total debt to my wonderful trainees, my outstanding lab and administrative assistants, and my great collaborators and other scientific colleagues; it was they who Shepard’d my ideas to fruition at the workbench. Most of all, I am grateful for my precious partner-in-life and wife (Sally), my loving children (Bert Jr, Becky, Sally Jr), and my extended family and friends. It was they who supported me and freed my mind to wander relentlessly, and sometimes recklessly, into the nuclear scientific unknown. When viewed in this light, my discoveries appear to be smaller and only part of a much larger scenario of the academic world. In short, our lab’s work and final biological messages were meant to be delivered to the world, eventually, and I just was lucky to be the messenger. (Thanks, Eime).

## Conflict of interest

The author declares no conflicts of interest with the contents of this article.
